# Cancer stem cells in melanoma

**DOI:** 10.3332/ecancer.2008.114

**Published:** 2008-12-01

**Authors:** C Regenbrecht, Y Welte, R Hugel, U Trefzer, FO Losch, J Adjaye, P Walden

**Affiliations:** 1 Max Planck Institute for Molecular Genetics, Berlin, Germany; 2 Clinical Research Group on Tumour Immunology, Department of Dermatology, Charite-Universitatsmedizin, Berlin, Germany

## Abstract

The identification of cancer stem cells in various malignancies led to the hypothesis that these cells have the exclusive ability of self-renewal, contribute to the plasticity of the tumours and may be the cause for ineffective cancer therapies. Several markers of melanoma stem cells have been described in recent studies including CD133, CD166, Nestin and BMI-1. Further studies are necessary to identify, better define and understand the origin and function of cancer stem cells. If confirmed that cancer stem cells play an important role in malignancy, therapeutic strategies may need to be redirected towards these cells to circumvent the failure of conventional therapies.

## Results

Using three different approaches we investigated ten low-passage melanoma cell lines established from metastatic lesions of melanoma patients from Charite-Universitatsmedizin, Berlin for the existence of putative cancer stem cells.

**Figure 1:**
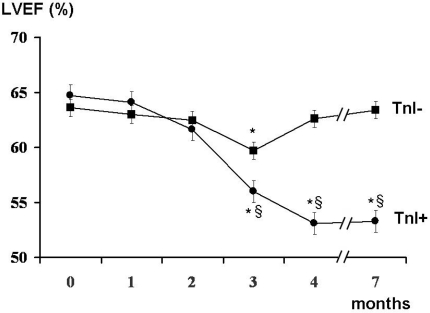
Change of phenotype of a melanoma cell line after cultivation in ES medium. Within ten days, originally adherent, large, elongated cells (A) formed clusters of non-adherent, small, round-shaped cells (B and C). Under ES culture condition, this phenotype remained stable for at least 8 weeks, whereas in standard medium an adherent monolayer was maintained.

**Figure 2:**
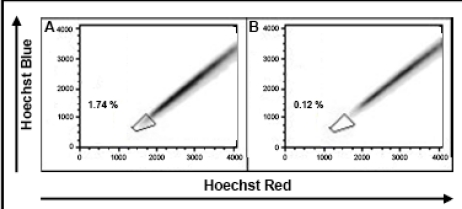
Identification of cancer stem cells as side population via Hoechst dye staining. Example of a melanoma cell line containing 1.7% putative cancer stem cells (A). Inhibition of ABC transporters that efflux the Hoechst dye led to a decrease of the side population (B).

**Figure 3:**
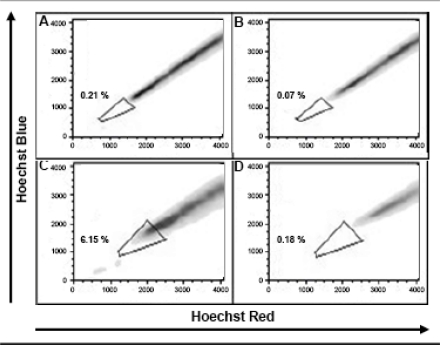
Enrichment of cancer stem cells under ES culture condition. Melanoma cell lines cultivated in standard medium (A) and ES medium (C) were incubated with Hoechst 33342 and analyzed for Hoechst dye emission. Whereas in standard medium no side population of putative cancer cells were detected, a side population of more than 6% emerged under ES cell condition Verapamil control (B and D).

**Figure 4:**
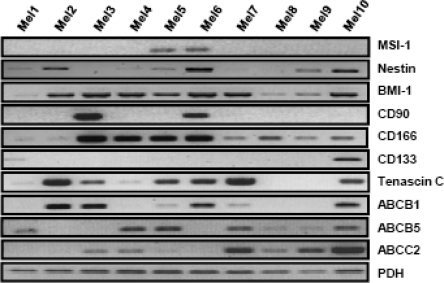
Expression pattern of putative cancer stem cell markers in ten melanoma cell lines. Analysis of embryonic stem cell markers BMI-1, CD90, CD166 and CD133; neural stem cell markers MSI-1 and Nestin as well as the in-melanoma over-expressed genes Tenascin C and ABC transporters by RT-PCR. All markers showed a heterogeneous expression pattern in these melanoma cell lines. Only BMI-1 and CD166 were expressed in all analysed melanoma cell lines.

1. Cultivation of melanoma cell lines in embryonic stem (ES) cell medium containing FGF2. This led to propagation of non-adherent spheres of small, round-shaped cells in six out of the ten cell lines within ten days (Figure 1B–C). The depicted phenotype remained stable for at least eight weeks, whereas in standard medium all cells were adherent, elongated and large (Figure 1A).
2. Identification of cancer stem cells as side population via staining with the DNA-binding dye Hoechst 33342 [3], which is based on the increased efflux of Hoechst by cancer stem cells relating to higher expression levels of ABC transporters. Under standard culture conditions nine melanoma cell lines exhibited a side population of such dye-low cells (Figure 2A), which decreased from ∼1% to nearly 0% after blocking of ABC transporters with Verapamil (Figure 2B). In one melanoma cell line, nearly no side population was detected under standard cell culture condition (Figure 3A). However, after four weeks of cultivation in ES medium, cancer stem cells were enriched as an enlarged side population of 6.15% of the cells (Figure 3C).
3. Identification of cancer stem cells by analyses of melanoma cell lines for the expression of known stem cell and cancer stem cell markers. The expression of all cancer stem cell markers was extremely heterogeneous among the tested samples (Figure 4, Table 1). By RT-PCR only the activated leukocyte cell adhesion molecule CD166 [1] and the B cell specific Moloney murine leukaemia virus integration site BMI-1 [2] both involved in melanoma metastasis were detected in all analyzed melanoma cell lines.

## Conclusion

There is good evidence supporting a shift of paradigms in understanding cancer, but still the origin of cancer stem cells and their defining properties remain elusive. Only by combining approaches from stem cell and cancer research, it may become possible to identify, characterize and use these cells in future cancer treatment.

## Figures and Tables

**Table 1:**
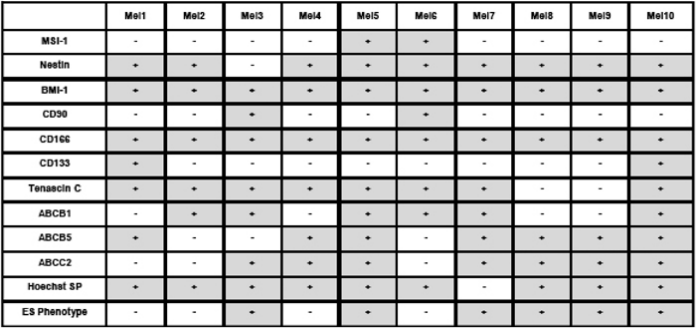
Summary of the expression pattern of different putative cancer stem cell markers in ten melanoma cell lines, the presence of a Hoechst side population and cells with ES phenotype
