# Erratum: Cancer stem cells in melanoma

**DOI:** 10.3332/ecancer.2012.249

**Published:** 2012-03-19

**Authors:** C Regenbrecht, Y Welte, R Hugel, U Trefzer, FO Losch, J Adjaye, P Walden

*ecancer***2** 114 (2008)

An incorrect figure was included as figure 1, please see the corrected figure below.

**Figure 1:**
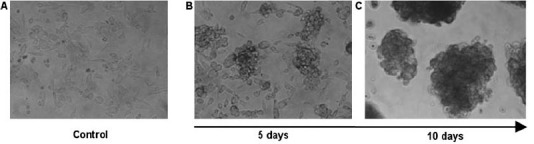
Change of phenotype of a melanoma cell line after cultivation in ES medium. Within ten days, originally adherent, large, elongated cells (A) formed clusters of non-adherent, small, round-shaped cells (B and C). Under ES culture condition, this phenotype remained stable for at least 8 weeks, whereas in standard medium an adherent monolayer was maintained.

